# Research progress of epithelial-mesenchymal transformation-related transcription factors in peritoneal metastases

**DOI:** 10.7150/jca.98409

**Published:** 2024-08-19

**Authors:** Lei Wang, Xiaobei Peng, Chang Ma, Lin Hu, Min Li, Yuhong Wang

**Affiliations:** 1 Institute of Biology and Medical Sciences, Soochow University, Suzhou 215123, China.; 2 Suzhou Medical College of Soochow University, Soochow University, Suzhou 215123, China.; 3 State Key Laboratory of Radiation Medicine and Protection, School of Radiation Medicine and Protection & School for Radiological and Interdisciplinary Sciences (RAD-X), Collaborative Innovation Center of Radiation Medicine of Jiangsu Higher Education Institutions, Soochow University, Suzhou 215123, Jiangsu, China.; 4 Department of Pathology, The First Affiliated Hospital of Soochow University, Suzhou, 215000, China.

**Keywords:** Peritoneal metastasis, Epithelial-mesenchymal Transition, Transcription factor

## Abstract

Metastasis is the leading cause of mortality in patients with malignant tumors, particularly characterized by peritoneal metastases originating from gastric, ovarian, and colorectal cancers. Regarded as the terminal phase of tumor progression, peritoneal metastasis presents limited therapeutic avenues and is associated with a dismal prognosis for patients. The epithelial-mesenchymal transition (EMT) is a crucial phenomenon in which epithelial cells undergo significant changes in both morphology and functionality, transitioning to a mesenchymal-like phenotype. This transition plays a pivotal role in facilitating tumor metastasis, with transcription factors being key mediators of EMT's effects. Consequently, we provide a retrospective summary of the efforts to identify specific targets among EMT-related transcription factors, aimed at modulating the onset and progression of peritoneal metastatic cancer. This summary offers vital theoretical underpinnings for developing treatment strategies against peritoneal metastasis.

## Introduction

Gastric cancer (GC) is a malignancy of epithelial origin that develops within the stomach and stands as one of the most prevalent cancers in China. Peritoneal metastasis emerges as a frequent occurrence in GC, serving as a common site for both metastatic spread and disease recurrence 1, 2, with a median survival of 3 to 6 months following peritoneal metastasis 3. Ovarian cancer (OC) stands as the most fatal malignancy affecting the female reproductive system, characterized by a startling 70% of patients presenting with extensive intra-abdominal dissemination upon their initial diagnosis. This spread complicates surgical removal and leads to recurrent relapses, significantly impacting patient survival outcomes 4, 5. Global cancer statistics by Siegel *et al.* indicates a 5-year survival rate of less than 20% for these patients 5. Colorectal cancer (CRC) stands as a prevalent malignancy within the digestive tract. Both international and national epidemiological statistics presented by Siegel *et al.* and the team of Academician He Jie indicate that CRC has a high incidence and mortality rate 1, 6. The incidence of peritoneal metastasis in CRC ranges from 4% to 19% 7-9, with a median survival of approximately 6 months 10, 11. These findings highlight the poor prognosis associated with common peritoneal metastatic cancers.

Epithelial-mesenchymal transition (EMT) includes three distinct types: Type I EMT, originally recognized as a crucial mechanism in early embryonic morphogenesis, is involved in critical developmental phases such as gastrulation, the formation of the neural crest, and cardiac morphogenesis. Type II EMT primarily functions in inflammatory responses, including wound healing, tissue regeneration, and fibrosis. Type III EMT, critical for cancer metastasis, involves changes in tumor cell heterogeneity and the local microenvironment, leading some epithelial tumor cells at the primary site to transition toward a mesenchymal phenotype 12-14. Through this transition, these cells gain enhanced invasive and migratory capabilities, breaking free from the primary site and entering the circulatory system to initiate the early stages of malignant metastasis 15. Current research on EMT primarily focuses on its role in cancer metastasis. However, studies on EMT in peritoneal metastatic cancer are sparse, considering its low incidence yet high mortality. This review aims to discuss the advances in research on EMT-related transcription factors involved in the molecular regulatory mechanisms and interventions at multiple steps of peritoneal metastatic cancer.

## Epithelial-mesenchymal transition (EMT)

The primary pathways of peritoneal metastatic cancer include: 1) Downregulation of different adhesion molecules on the surface of tumor cells, resulting in the detachment of tumor cells; 2) Detached cancer cells acquire more mesenchymal characteristics, enhancing their invasiveness and resistance to apoptosis; 3) Detached cancer cells invade the peritoneum via the mesothelial/lymphatic pathways; 4) Cancer cells invading the peritoneum predominantly aggregate in milky spots; 5) Peritoneal-implanted cancer cells proliferate extensively 16, 17. In peritoneal metastatic cancer, epithelial-mesenchymal transition (EMT) primarily functions through transcriptional regulation, which is driven by EMT itself.

Transcription factors, a set of proteins, specifically bind to sequences located upstream of a gene's 5' region. This binding ensures that the target gene is expressed with particular intensity, timing, and spatial distribution 18. EMT-related transcription factors primarily include the Snail family, Twist family, and zinc-finger E-box binding (ZEB) transcription factor family. Other families involved are the Kruppel-like factor family, forkhead box family, SRY-related high-mobility group, RUNX family, and GATA family. This review will sequentially discuss the role of these EMT-related transcription factors in peritoneal metastatic cancer.

### Snail Family Transcription Factors

The Snail family genes encode transcription factors with zinc finger structures and are widely regarded as typical EMT-related transcription factors due to their involvement in regulating multiple physiological levels through binding to downstream genes. The Snail family primarily includes two members: Snail1 (commonly referred to as Snail) and Snail2 (also known as Slug) 19. All members of this family encode transcriptional repressors that possess a highly conserved C-terminal domain, containing 4-6 C2H2-type zinc fingers, which bind to the E-box motifs (5'-CANNTG-3') of target gene promoters 20.

Current perspectives suggest that fibrin deposits on the peritoneal surface serve as a breeding ground for cancer dissemination in cancer patients 21; significant upregulation of Snail is observed in both gastric cancer and ovarian cancer peritoneal metastases 22, 23. Mechanistically, the MUC4 protein promotes peritoneal metastasis by upregulating Snail 24; miR-22 induces extracellular matrix (ECM) remodeling and EMT by upregulating MMP14 and Snail, thereby facilitating peritoneal and lung metastasis in gastric cancer 25. β-hCG and estrogen-related receptor alpha (ERRα) mediate ovarian cancer peritoneal metastasis through EMT and Snail 26, 27. CST1 Promotes the EMT Process in Gastric Cancer by Upregulating Snail 28. At the same time, strategies aimed at targeting Snail in peritoneal metastatic cancer reveal that melatonin suppresses EMT progression, diminishes Snail expression, and enhances endoplasmic reticulum (ER) stress, thereby hindering gastric cancer peritoneal metastasis 29. The novel aryl hydrocarbon receptor inhibitor, Biseugenol, activates Calpain-10 and inhibits Aryl hydrocarbon receptor, thus inducing ER stress and obstructing gastric cancer peritoneal metastasis 30. An ethyl acetate extract from daylily (Hemerocallis fulva), COE, inhibits gastric cancer peritoneal metastasis by suppressing the HSP27-mediated NF-κB/Snail signaling pathway 31.

Research on Slug's role in peritoneal metastatic cancer shows that ARL4C promotes gastric cancer peritoneal metastasis by activating Slug 32; CEACAM6 enhances EMT and Slug expression via the PI3K/AKT signaling pathway to promote gastric cancer peritoneal metastasis 33; and miR-203 targets the ERK1/2/Slug/E-cadherin signaling pathway to inhibit gastric cancer peritoneal metastasis 34.

These investigations emphasize the crucial role of the Snail family in facilitating peritoneal metastasis, especially in gastric and ovarian cancers. Research focused on targeting the Snail family for therapeutic interventions in peritoneal metastasis is making steady progress.

### Twist Family Transcription Factors

Twist, a highly conserved member of the basic helix-loop-helix (bHLH) transcription factor family, is pivotal in embryonic development and tissue morphogenesis. Furthermore, Twist aids in the advancement of malignant tumors primarily by transcriptionally regulating and facilitating the epithelial-mesenchymal transition (EMT), which promotes cancer progression 35. Twist is a highly conserved protein with 96% amino acid sequence homology between mice and humans, and its DNA binding domain is 100% conserved across different species, containing the E-box binding DNA sequence: 5'-CANNTG-3' 36, 37.

Current research on Twist in peritoneal metastatic cancer indicates that Twist promotes peritoneal metastasis in ovarian cancer 38. Mechanistically, asparagine endopeptidase (AEP) upregulates Twist to promote peritoneal metastasis in gastric cancer 39; EG-1 upregulates eIF4E-mediated MMP-9 and Twist to facilitate gastric cancer metastasis 40; TrkB promotes EMT and Twist-enhanced peritoneal metastasis and apoptosis resistance in ovarian cancer 41. Additionally, interventions targeting Twist in peritoneal metastatic cancer have been studied by Huang *et al.*, who demonstrated that dextran sulfate (DS) inhibits hypoxia-inducible factor-1α (HIF-1α) expression, suppresses TGF-β-mediated EMT in gastric cancer cells, and thus inhibits gastric cancer peritoneal metastasis 42.

In summary, Twist significantly contributes to peritoneal metastasis in tumors, with research primarily centered on gastric and ovarian cancers. Efforts to target Twist for inhibiting tumor peritoneal metastasis are progressing, underscoring its potential as a therapeutic target in these aggressive forms of cancer.

### Zinc Finger E-box-Binding (ZEB) Transcription Factors

The zinc finger E-box binding protein (ZEB) family is instrumental in the development and progression of cancers. This family comprises two primary members: ZEB1 (also known as TCF-8 or δEF1) and ZEB2 (also known as SIP1) 43. Both genes belong to the C2H2 type zinc finger protein family, characterized by a central homology domain along with four N-terminal zinc fingers and three C-terminal zinc fingers 44. Research indicates that both ZEB1 and ZEB2 bind to the E-box consensus sequence 5'-CANNTG-3' on the CDH1 promoter. They exert suppression of CDH1 expression by recruiting repressive protein complexes such as C-terminal binding proteins (CtBP), Polycomb proteins, CoREST, and the SWI/SNF chromatin remodeling complex BRG1^45^.

Research on ZEB1 in peritoneal metastatic cancer indicates that patients with high expression of ZEB1 in gastric cancer peritoneal lavage fluids have poor prognosis 46. Gastric cancer tissues with high ZEB1 expression also show an increased risk of peritoneal metastasis 47. A gastric cancer stem cell peritoneal metastasis model demonstrated upregulation of ZEB1 through immunofluorescence 22. Molecular investigations have revealed that subtypes of TGF-β (TGF-β1, TGF-β2, and TGF-β3) upregulate ZEB1 expression, promoting ovarian cancer peritoneal metastasis 48. Tissue transglutaminase (TG2) activates NF-κB, which in turn activates ZEB1 to promote ovarian cancer peritoneal metastasis 49. miR-34b-5p Inhibits Peritoneal Metastasis of Endometrial Cancer by Targeting ZEB1 50. Targeted studies on ZEB1 have shown that FOXM1 and EGFR/ERBB2 can upregulate ZEB1, promoting ovarian cancer peritoneal metastasis, while combined treatment with lapatinib (a dual kinase inhibitor of EGFR/ERBB1 and ERBB2) and thiostrepton (an inhibitor of FOXM1) can reverse this mechanism and inhibit ovarian cancer peritoneal metastasis 51.

Research on ZEB2 in peritoneal metastatic cancer shows that ZEB2 promotes peritoneal metastasis of high-grade serous ovarian cancer by regulating cancer stem-like cells 52. Additionally, the interaction of gastric cancer cells with nearby cancer-associated fibroblasts (CAFs) leads to the production of IL-33. This cytokine, via ST2L-dependent activation of the ERK1/2-SP1-ZEB2 pathway, triggers epithelial-mesenchymal transition (EMT), subsequently increasing the migratory and invasive properties of the gastric cancer cells 53.

These findings demonstrate the ZEB family's role in promoting peritoneal metastatic cancer, with high expression of ZEB1 in ascites associated with poor prognosis. The focus of related research has predominantly been on gastric and ovarian cancers' peritoneal metastasis.

### Other EMT-related transcription factors

In addition to the established EMT-related transcription factors, recent research has identified an increasing number of other transcription factors that play significant roles in the EMT process. This section will focus on these other EMT-related transcription factors that facilitate the progression of peritoneal metastatic cancer.

The Kruppel-like factor (KLF) family consists of transcription regulators with C2H2 zinc finger structures and includes 17 members, named KLF1 through KLF17 54. Studies have shown that EIF5A2 promotes cancer cell stemness and peritoneal metastasis through the E2F1/KLF4 pathway 55. Multi-omic analyses of ascites from gastric cancer patients with peritoneal metastasis revealed active super-enhancers at the ELF3, KLF5, and EHF gene loci 56. Interestingly, existing research on KLF12 has demonstrated its role in promoting the malignant progression of both pancreatic and ovarian cancers 57-60, yet a study by Celia S. L. and colleagues revealed that high expression of miR-141 in ovarian cancer inhibits KLF12 61, thus promoting peritoneal metastasis, indicating that the role of KLF12 requires further exploration.

The forkhead box (FOX) transcription factors are characterized by a highly conserved winged helix DNA-binding domain and consist of 50 human genes 62. Members of the FOX family associated with EMT include FOXA1, FOXA2, FOXC1, FOXC2, FOXD2, FOXF1, FOXF2, FOXG1, FOXK1, FOXM1, FOXN2, FOXO3A, FOXQ1, FOSL1, and FOSL2. STC1 promotes ovarian cancer peritoneal metastasis via the FOXC2/ITGB6 signaling axis 63; FOXF1 promotes peritoneal metastasis of colorectal cancer through transcriptional activation of SNAI 64; FOXM1 regulates ZEB1 to enhance ovarian cancer peritoneal metastasis 51; Additionally, exosomes from omental adipose tissue-derived mesenchymal stem cells have been shown to exacerbate ovarian cancer peritoneal metastasis through FOXM1 and associated Cyclin F, KIF20A, and MAPK signaling pathways, providing new insights into therapeutic interventions 65. BTF3 induces gastric cancer peritoneal metastasis by modulating FOXM1 and the JAK2/STAT3 signaling pathway 66.

The SRY-related HMG Box (SOX) proteins are a subgroup within the high mobility group (HMG) proteins. Research indicates that the SOX family genes play significant roles in the proliferation, migration, invasion, and metastasis of various cancer cells 67. The SOX family includes numerous transcriptional regulators that mediate DNA binding through the HMG domain. This domain comprises approximately 79 amino acid residues and features six core sequences, WWCAAW (W = A/T) 67, 68. Current studies have identified SOX4, SOX9, and SOX11 as transcription factors related to epithelial-mesenchymal transition (EMT), with a particular focus on SOX9 in the context of peritoneal metastasis-related cancers. SOX9 transcriptionally activates S100P, which suppresses the RAGE/ERK signaling pathway and promotes EMT, thus aiding the peritoneal spread of colorectal cancer 69. Additionally, the p70S6 kinase (p70S6K)-miR-145 pathway promotes peritoneal metastasis in ovarian cancer by targeting Twist and Sox9 70.

The GATA family of transcription factors is a group of evolutionarily conserved zinc finger proteins that play multifaceted roles in cell differentiation and organ development during the early stages across various tissues 71 This family primarily consists of six transcription factors, GATA1 through GATA6, with initial functional studies focusing on hematopoiesis and cardiac development 72. However, their functions and expression patterns extend well beyond these tissues. Recent research has discovered that GATA6-AS1 inhibits the epithelial-to-mesenchymal transition (EMT) of pancreatic cancer under hypoxic conditions by regulating the stability of SNAI1 mRNA 73.

Additional transcription factors associated with epithelial-mesenchymal transition (EMT) have been implicated in promoting peritoneal metastasis in cancer. Notably, OVOL2 is significantly overexpressed in a peritoneal metastasis tumor model constructed with gastric cancer stem cells 22. Similarly, WT1 is highly expressed in the peritoneal metastasis model of ovarian low-grade serous carcinoma 74, 75, and shows a sensitivity of 93% in diagnosing metastatic ovarian cancer 76; SMAD3, as a key factor in the TGF-β signaling pathway, plays an important role in the peritoneal metastasis of colorectal cancer 77.

In summary, other transcription factors related to epithelial-mesenchymal transition (EMT) primarily function to promote the occurrence and progression of peritoneal metastatic cancer. In the future, research on EMT-related transcription factors in peritoneal cancer will not be limited to those already identified but will also include investigations into the roles and molecular mechanisms of emerging EMT-related transcription factors in peritoneal metastasis.

## Conclusion

Peritoneal metastasis is among the prognostically poorer groups in cancer patients. Taking gastric cancer peritoneal metastasis as a typical example, its early stages are predominantly characterized by metastasis, lacking effective means for early detection. As a result, a majority of patients receive diagnoses at advanced stages. After onset, peritoneal metastasis in gastric cancer progresses swiftly, drastically diminishing patient survival rates. The underlying mechanisms involve the gastric cancer cells' ability to remodel the extracellular matrix, induce the transformation of normal peritoneal cells into a tumor phenotype, induce angiogenesis around peritoneal colonization sites, regulate the immune function of the tumor microenvironment, and alter the epithelial and mesenchymal phenotypes of tumor cells. Consequently, identifying molecular targets and corresponding interventions for peritoneal metastasis remains a focus for clinical researchers.

Epithelial-mesenchymal transition (EMT) reveals that tumor-related epithelial cells undergo a series of changes and transformations to convert into mesenchymal phenotype tumor cells, depending on the tissue and signaling environment. Meanwhile, the process of mesenchymal-epithelial transition (MET), although not as evident as EMT, demonstrates the reversibility of these transformations. This review retrospectively summarizes the roles and molecular mechanisms of EMT-related transcription factors in peritoneal metastatic cancer. It is noteworthy that most studies on EMT-related transcription factors are conducted in a cell/tissue-type specific manner, and we still lack sufficient information to understand whether their functions and molecular mechanisms are consistent across different cell/tissue types or even in different environments. Therefore, further exploration into the similarities and differences in the roles of EMT-related transcription factors across various cells, tissues, and environments is essential to better elucidate their specific functions and mechanisms.

Additionally, future research should focus more on the roles of other known transcription factors in peritoneal metastasis and explore the roles of emerging EMT-related transcription factors in this context. Finally, the function of EMT is not entirely mediated by transcription factors; it also includes EMT-mediated signal transduction and EMT-related non-coding RNAs. Among these, the Transforming Growth Factor β (TGF-β) is one of the most extensively studied signaling pathways, regulating the initiation and progression of EMT through various mechanisms. Additionally, receptor tyrosine kinases (RTK), Wnt, Notch, Hedgehog, Hippo, and other signaling pathways also play critical roles in EMT regulation 78. The interconnections among these signaling pathways collectively regulate the complex process of EMT, thereby affecting the metastatic and invasive abilities of tumor cells 79.

Research indicates that non-coding RNAs, such as the miR-200 family and the miR-34 family, exhibit significant tumor metastasis inhibition functions by regulating transcription factors like ZEB and Snail 78. Moreover, inflammatory mediators in the tumor microenvironment, such as cytokines (e.g., IL-1, IL-6, IL-8, and TNF-α) and chemokines (including CCL5, SDF-1, CCL2, and CCL7), also promote the EMT process 80. They regulate the metastatic and invasive abilities of cancer cells by binding to specific membrane receptors and activating internal signaling pathways 81.

Therefore, novel therapeutic strategies targeting these signaling pathways, non-coding RNAs, and inflammatory mediators have significant clinical prospects. They can effectively hinder the progression of metastatic cancer, reduce cancer recurrence, and prevent the development of treatment resistance 82. For example, inhibitors targeting TGF-β receptor kinases combined with cytotoxic drugs such as paclitaxel can not only effectively inhibit the EMT process in breast cancer cells but also reduce their metastatic potential in the lungs 78. Additionally, targeting inflammatory mediators with natural anti-inflammatory compounds and promoting the use of lipid-soluble drugs to block tumor metastasis is another potential strategy 83.

This is also the work our team plans to undertake next. By comprehensively summarizing the current functions and mechanisms of EMT in peritoneal metastasis can we better explore the potential of MET and provide guiding suggestions for identifying potential targets to reverse peritoneal metastasis.

## Figures and Tables

**Figure 1:**
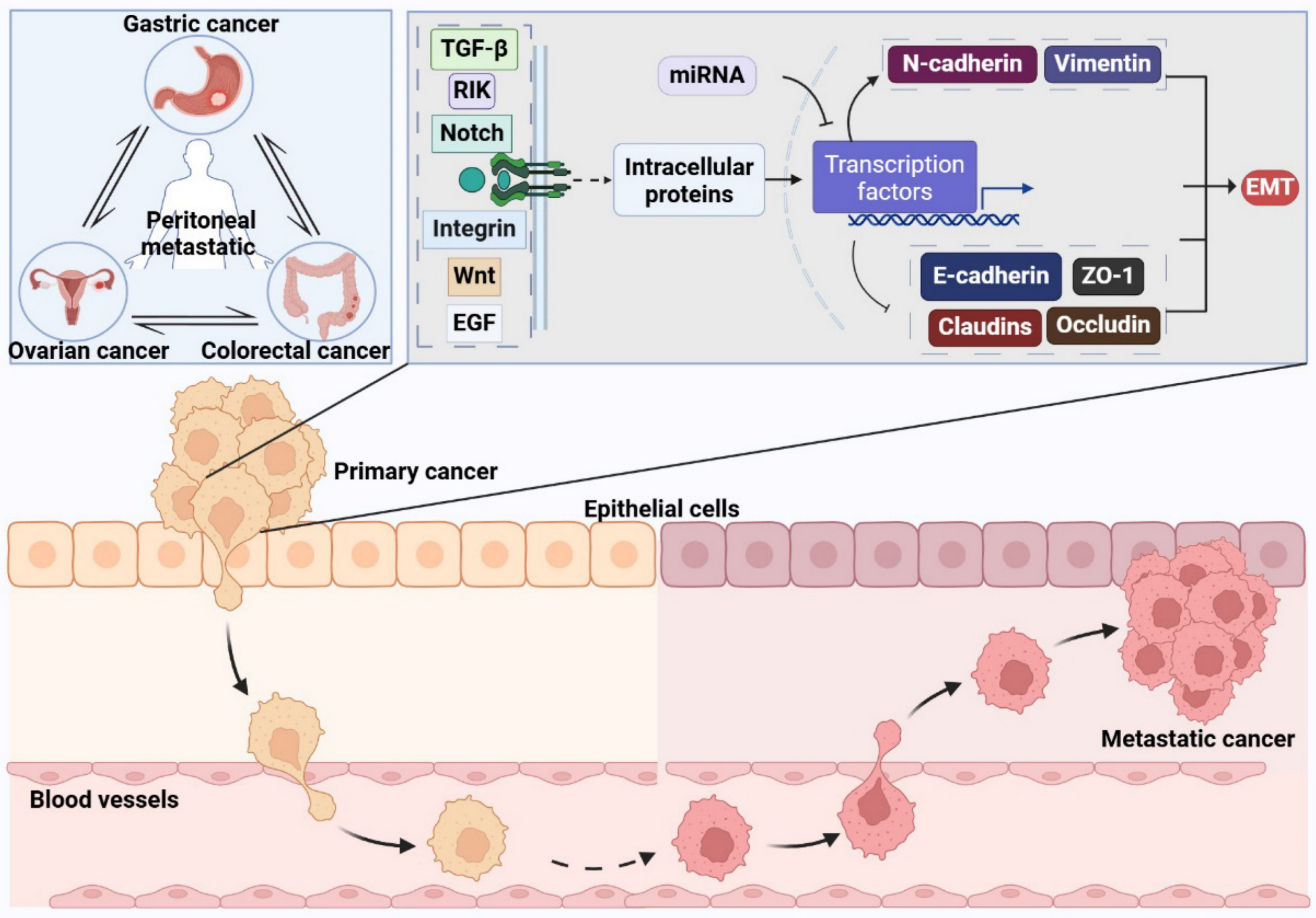
** The Mechanisms of Epithelial-Mesenchymal Transition.** The process of inducing EMT involves a variety of extracellular signaling molecules, intracellular proteins, and the miRNA group, including Transforming Growth Factor-beta (TGF-β), Wnt, Epidermal Growth Factor (EGF), integrins, Notch, and the associated kinase RIK. These components trigger a signaling cascade upon binding to specific receptors situated on the cell membrane. Crucial steps in this signaling pathway encompass the activation of diverse downstream proteins, which subsequently modulate the activity of particular transcription factors. Upon activation, transcription factors such as Snail, Slug, Twist, and members of the ZEB family prompt the reduction in cell surface adhesion molecules like E-cadherin, ZO-1, claudins, and occludin. This change leads to the disruption of tight intercellular junctions and diminished cell adhesion. Concurrently, the transcription factors also stimulate the elevation of mesenchymal markers such as N-cadherin and vimentin, thereby facilitating the acquisition of mesenchymal traits by the cells.

**Figure 2:**
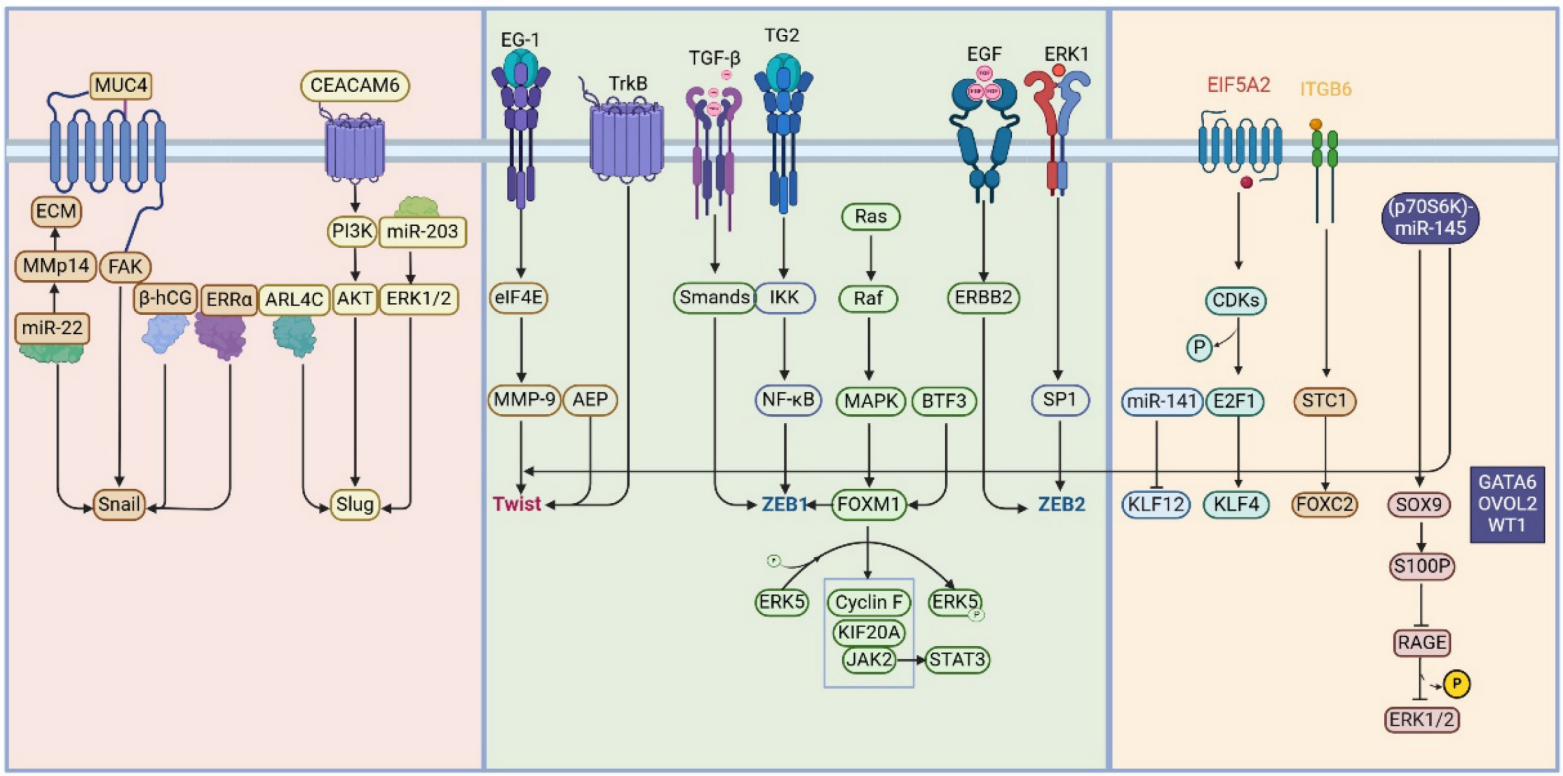
** Transcription Factors and Signaling Pathways in the Process of EMT.** Throughout the process of epithelial-mesenchymal transition (EMT), regulation encompasses a diverse array of transcription factors and signaling pathways. The principal transcription factors encompass the Snail family, Twist family, and the zinc-finger E-box binding (ZEB) transcription factor family. Furthermore, this regulatory process involves the Kruppel-like factor (KLF) family, Forkhead box (Fox) family, SRY-related HMG-box (SOX) family, RUNX family, and GATA family. During EMT, a multitude of extracellular and intracellular proteins activate various intracellular signaling pathways, thereby modulating the expression of these transcription factors and facilitating the progression of EMT.

**Table 1:** Mechanisms of action of translational factors in peritoneal metastasis

Transcription Factor	Target gene function		Interaction signal pathways	Ref
	Upregulation	Downregulation		
Snail	N-cadherin; Vimentin ERK1/2; Akt; MMP9; MMP15; MMP2; MMP14; TWIST; ZEB1; ZEB2	E-cadherin; Claudins; Occludin	FAK; TGFβ/SMAD3; Wnt/β-catenin; Notch; PI3K/AKT; NF-Κb; ATK/MAPK; EGF; FGF; RTKs	21-31
Slug	N-cadherin; Vimentin; MMP9	E-cadherin	ERK1/2/Slug/E-cadherin; PI3K/AKT	32-34
Twist	N-cadherin; MMPs; Vimentin; β-catenin	E-cadherin; E-cadherin; Claudins; Occludin; ERK1/2; Akt; MMP9	FAK; MAPK; PI3K/AKT; Wnt/β-catenin	35-42
ZEB1	N-cadherin; MMPs; Vimentin	E-cadherin; ZO1 Claudins; Occludin	TGFβ/SMAD3, Wnt/β-catenin; RAS/MAPK	46-51
ZEB2	N-cadherin; Vimentin	E-cadherin	ERK1/2/SP1/ZEB2	52, 53
KLF12	ISG15; miR-137	E-cadherin	Wnt/β-catenin	57-61
FOXC2	Vimentin; N-cadherin	E-cadherin	TGFβ/SMAD3; MAPK/AKT	
FOXF1	Fibronectin; N-cadherin; Snail; Vimentin	E-cadherin; Claudin1; Occludin; ZO1	Unknown	63
FOXM1	N-cadherin; ZEB2 KLF20A; GFR; ERBB2	E-cadherin; ZRB1	JAK2/pSTAT3	64
SOX9	S100p; SNAIL2; N-cadherin	Unknown	BMPs; PKA; PI3K/AKT; RAGE/ERK	69, 70
GATA6	N-cadherin; MMP1	Fibronectin; N-cadherin; SNAI1	Wnt/β-catenin	72, 73
OVOL2	Vimentin; α-SMA	E-cadherin	Wnt/β-catenin	22
WT1	Unknown	Unknown	Wnt/β-catenin	74-76
SMAD3	ANGPTL4	Unknown	TGF-β1/SMAD3/ANGPTL4	77

**Table 2:** Diagnostic and Therapeutic Value of Transcription Factors

Transcription Factor	Therapeutic value	Diagnostic value
Snail Famliy	Ovarian cancer 23 Gastric cancer 22 Lung cancer 25	Gastric cancer 29, 31
Twist Famliy	Ovarian cancer 41 Gastric cancer 39, 40	Gastric cancer 42
ZEB Famliy	Gastric cancer 22, 47 Ovarian cancer 48, 49 Endometrial cancer 50	Ovarian cancer 51
KLF Famliy	Gastric cancer 56, 57 Pancreatic cancer 58 Ovarian cancer 55	Ovarian cancer 61
FOX Famliy	Ovarian cancer 63, 65 Colorectal cancer 64 Gastric cancer 66	Gastric cancer 65
SOX Famliy	Colorectal cancer 69 Ovarian cancer	/
GATA Famliy	Pancreatic cancer 73	/
OVOL2	Gastric cancer 22	/
WT1	Ovarian serous carcinoma 76	/
SMAD3	Colorectal cancer 77	/
